# Supplementary material for: Modeling lung perfusion abnormalities to explain early COVID-19 hypoxemia
Source: Nat Commun. 2020 Sep 28;11:4883. doi: 10.1038/s41467-020-18672-6 (PMC7522238; doi:10.1038/s41467-020-18672-6)
Supplement: Supplementary file 1 — Supplementary Information [file 41467_2020_18672_MOESM1_ESM.pdf]

Modeling Lung Perfusion Abnormalities to Explain Early COVID-19 Hypoxemia

Supplementary Information

Jacob Herrmann[1]*, Vitor Mori[2], Jason H.T. Bates[2], Béla Suki[1]

[1]Dept. of Biomedical Engineering, Boston University, Boston, MA, USA

[2]Dept. of Medicine, University of Vermont, Burlington, VT, USA

* Corresponding Author

Supplementary Discussion

Consequences of perfusion defect and perfusion defect

To accompany Figure 4 in the main text, a more comprehensive analysis of the contribution of perfusion defect to hypoxemia in the model is provided herein. In this supplementary analysis, two scenarios are explored: Supplementary Figs. 1 to 4 as well as Figure 4 in the main text pertain to the original modeling scenario of a patient upon admission, with F_{inj} ranging between 0% to 30%, inspired oxygen fraction $F_{\mathrm{i}}O_2$ = 21%, and mixed venous oxygen tension $P_{\mathrm{v}}O_2$ = 40 mmHg; Supplementary Figs. 5 to 9 describe the scenario of a critical care patient, with F_{inj} ranging up to 80%, $F_{\mathrm{i}}O_2$ = 40%, and $P_{\mathrm{v}}O_2$ = 25 mmHg. The model outcomes reported here include the ratio of arterial oxygen tension ($P_{\mathrm{a}}O_2$) to inspired oxygen fraction ($F_{\mathrm{i}}O_2$) in Supplementary Figs. 1 and 5; arterial oxygen saturation ($S_{\mathrm{a}}O_2$) in Supplementary Figs. 2 and 6; the calculated shunt fraction (F_{shu}) in Supplementary Figs. 3 and 7; the F_{shu}:F_{inj} ratio in Supplementary Fig. 8 (see also Figure 4 in the main text); and the pulmonary vascular resistance (PVR) in Supplementary Figs. 4 and 9.

Note that all of these outcomes—except PVR—are insensitive to the amount of perfusion defect when the perfusion defect is distributed uniformly in the model, without preference for injured vs. noninjured compartments. Generally, oxygenation improves when perfusion defect preferentially occurs in the injured lung, and worsens when perfusion defect occurs in the noninjured lung. Also note that $P_{\mathrm{a}}O_2$:$F_{\mathrm{i}}O_2$ ratios below 200 are not observed in the early stage model reported in the main text due to $F_{\mathrm{i}}O_2$ = 21% and $P_{\mathrm{v}}O_2$ = 40 mmHg (yielding at least $P_{\mathrm{a}}O_2$:$F_{\mathrm{i}}O_2$ = 190 mmHg even with 100% shunt). Modifying parameters to represent a critical care patient with $F_{\mathrm{i}}O_2$ = 40% and $P_{\mathrm{v}}O_2$ = 25 mmHg results in $P_{\mathrm{a}}O_2$:$F_{\mathrm{i}}O_2$ ratios as low as 63 mmHg, with especially low ratios when HPV is not normally functioning in the injured lung

compartments representing pulmonary shunt. Finally, note that perfusion defect results in increased PVR in nearly every simulated condition unless balanced by vasodilation in the reversed HPV model.

Effects of increased inspired oxygen fraction on hypoxemia

Patients with hypoxia are often administered supplemental oxygen, which can improve oxygenation of blood in regions with low ventilation:perfusion ratio. In our model, the inspired oxygen fraction (F_iO_2) was set between 21% and 100%. Similar to Figure 2 in the main text, the reversed HPV model was characterized by 72% reduction in vascular resistance of the injured regions, and baseline gravitational gradients of perfusion were set at 30%. In addition to the reference case without any perfusion defect (PD) or venous admixture (VA) in the noninjured lung compartments (Supplementary Fig. 10), we also included cases for PD = 60% representing thrombosis mediated perfusion defects, and VA = 37% representing severe ventilation-perfusion mismatching (Figure 7 in the main text).

Supplementary Figure 10 demonstrates that the model with pulmonary shunt in the injured lung compartments can exhibit both improving and worsening P_aO_2:F_iO_2 ratio as F_iO_2 increases, depending on the range of F_iO_2 and extent of injury. In general, P_aO_2:F_iO_2 and FiO2 are negatively correlated at low F_iO_2 and high F_{inj}, and positively correlated at high F_iO_2 and low F_{inj}. This may be attributed to the nonlinear nature of the oxygen-hemoglobin dissociation curve. Hemoglobin becomes fully saturated at blood oxygen tension around 100 mmHg, after which increasing F_iO_2 primarily increases the amount of dissolved oxygen gas. When shunted and oxygenated blood combine, a large amount of dissolved oxygen binds to unoccupied hemoglobin, and the oxygen tension of the mixed arterial blood is greatly reduced. Thus increasing F_iO_2 in a model of pulmonary shunt yields diminishing gains in P_aO_2, at least until the oxygenated blood is

sufficiently oxygen-rich such that all hemoglobin remain fully saturated after mixing with shunted blood. This can be observed in Supplementary Fig. 10: the turning point at which increasing F_iO_2 changes from negative to positive correlation with the P_aO_2:F_iO_2 ratio coincides with the F_iO_2 at which S_aO_2 reaches 100%. In cases with $F_{inj} > 50\%$, this may never occur even with $F_iO_2 = 100\%$.

Note that in Supplementary Fig. 10, the ratio F_{shu}:F_{inj} is insensitive to changes in F_iO_2. By comparison, F_{shu}:F_{inj} decreases with increasing F_iO_2 in the model of noninjured venous admixture due to ventilation-perfusion mismatching. This is because lung regions with low ventilation:perfusion ratio may in fact saturate hemoglobin and sufficiently oxygenate blood at very high F_iO_2 levels, since nitrogen in alveolar gas becomes almost entirely replaced by oxygen. Hypoxemia caused by lung regions of complete pulmonary shunt (i.e., with zero ventilation:perfusion ratio) is not responsive to changes in F_iO_2, as shown in Supplementary Fig. 10.

Interplay between mixed venous oxygen tension and hypoxic pulmonary vasoconstriction

Veno-venous extracorporeal membrane oxygen (V-V ECMO) removes deoxygenated blood from the systemic venous system, oxygenates the blood through a membrane, and returns the blood back into the systemic venous system. This invasive intervention enhances the oxygen content of mixed venous blood and may reduce ventilatory demand for critical care patients in need of respiratory support. Hypoxic pulmonary vasoconstriction (HPV) normally constricts pulmonary arterioles in lung regions with poor oxygenation, such as pulmonary shunt. If a patient with pulmonary shunt is placed on V-V ECMO, it is possible that normal HPV feedback may be destimulated due to increased mixed venous oxygen tension (P_vO_2). This could potentially result in increased perfusion to lung regions with poor ventilation and worsen ventilation-perfusion matching.

In our model, the potential interplay between HPV and V-V ECMO was explored using P_vO_2 ranging between 20 to 100 mmHg. The inspired oxygen fraction (F_iO_2) was set at 60%. Similar to Figure 2 in the main text, the reversed HPV model was characterized by 72% reduction in vascular resistance of the injured regions, and baseline gravitational gradients of perfusion were set at 30%. In addition to the reference case without any perfusion defect (PD) or venous admixture (VA) in the noninjured lung compartments, we also included cases for PD = 60% representing thrombosis-mediated perfusion defect, and VA = 37% representing severe ventilation-perfusion mismatching.

The results in Supplementary Fig. 11 demonstrate that normal HPV function in the injured lung compartment (blue) can compensate for a small amount of pulmonary shunt (F_{inj} = 17%), restoring a normal value of the $P_aO_2{:}F_iO_2$ ratio if there is no perfusion defect or ventilation-perfusion mismatching. By comparison, impaired HPV (red) results in $P_aO_2{:}F_iO_2 < 300$ mmHg for any $P_vO_2 < 60$ mmHg. When a large amount of perfusion defect is included in the model, total pulmonary vascular resistance increases, and a disproportionate amount of perfusion is redirected through the injured lung compartments. In this case, normal HPV function is unable to maintain $P_aO_2{:}F_iO_2 > 300$ mmHg, yet still maintains better oxygenation ($P_aO_2{:}F_iO_2$ between 100 to 200 mmHg) compared to impaired HPV ($P_aO_2{:}F_iO_2 < 100$ mmHg) at low $P_vO_2 < 60$ mmHg. However, when ventilation-perfusion mismatching yields 37% venous admixture in the noninjured lung compartments, HPV within the injured compartments is ineffective at preventing hypoxemia. Note that $F_{shu}{:}F_{inj}$ ratios are very high for the case with VA = 37%, even in the normal HPV model, because the calculated shunt fraction primarily reflects the venous admixture from the noninjured lung rather than the 17% injured fraction.

In all cases, the calculated shunt fraction increases in the normal HPV model as P_vO_2 increases, until the normal HPV and impaired HPV models are indistinguishable at $P_vO_2 > 70$ mmHg. If the benefit of V-V ECMO in the normal HPV model is assessed by arterial oxygenation alone (i.e., P_aO_2:F_iO_2 and S_aO_2), then it may seem as though increasing venous oxygenation results in apparently worse patient condition when P_vO_2 is in the range of 20 to 40 mmHg, and when there is no venous admixture in the noninjured lung. This drop in arterial oxygenation with increasing P_vO_2 may be attributed to relaxation of HPV. However as P_vO_2 further increases above 40 mmHg, arterial oxygenation metric rise and indicate improved patient condition. In the model of ventilation-perfusion mismatching with VA = 37%, V-V ECMO is beneficial at all levels of P_vO_2. Note although arterial oxygenation improves for $P_vO_2 > 40$ mmHg, there may be adverse consequences for hypercapnia if CO_2 is not sufficiently eliminated via ECMO. Also note that the calculated shunt fraction increases with increasing P_vO_2, but this metric may be misleading because the shunted blood is actually oxygenated.

Supplementary Figures

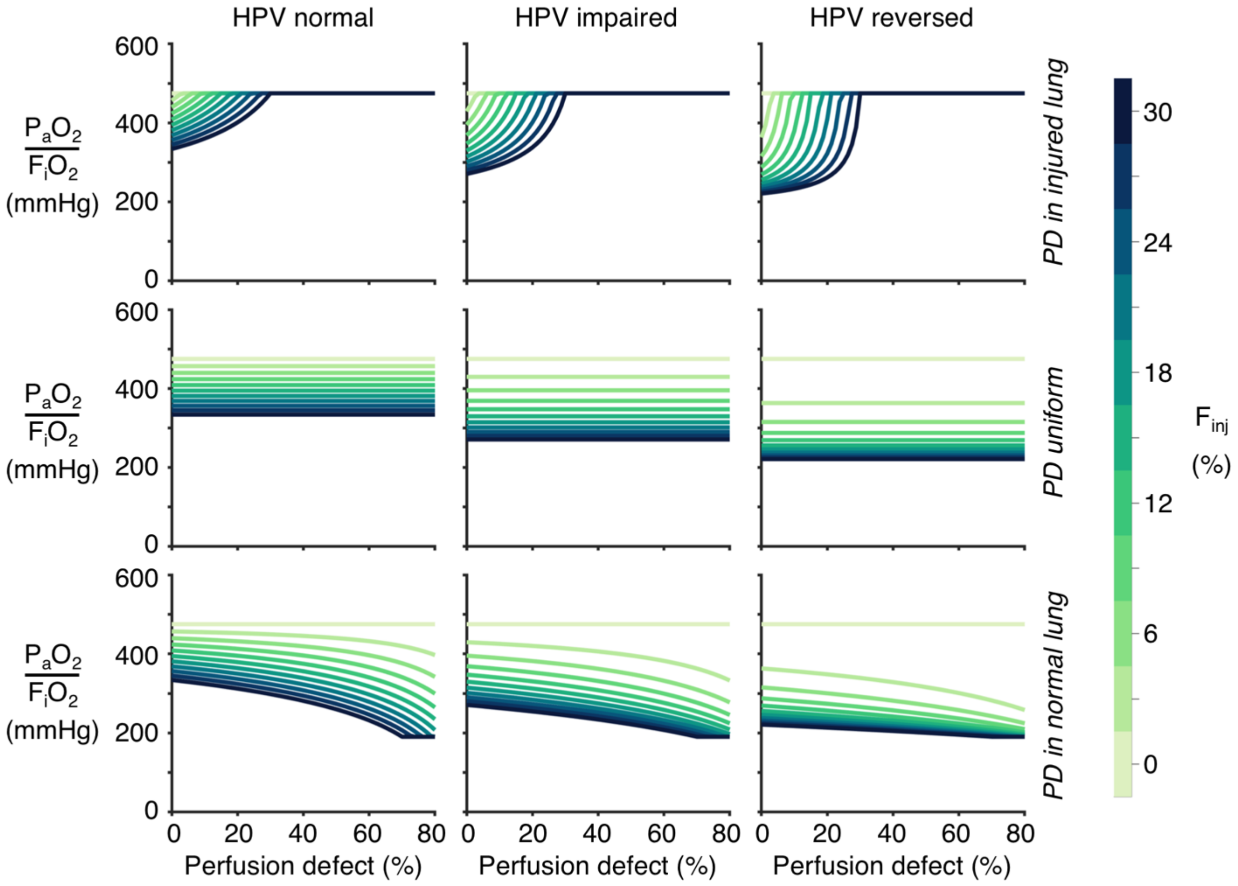

Supplementary Figure 1. Effect of perfusion defect (PD) on the ratio of arterial oxygen tension (P_aO_2) to inspired oxygen fraction (F_iO_2) in the early stage model. Columns correspond to alterations in hypoxic pulmonary vasoconstriction (HPV). Rows correspond to the distribution of perfusion defect. Color indicates the fraction of injured lung (F_{inj}). In all cases, F_iO_2 was 21%, mixed venous oxygen tension was 40 mmHg, baseline perfusion gradient was 30%, reversed HPV was modeled with 72% reduction of vascular resistance in injured regions, and there was no assumed ventilation-perfusion mismatching in the noninjured lung.

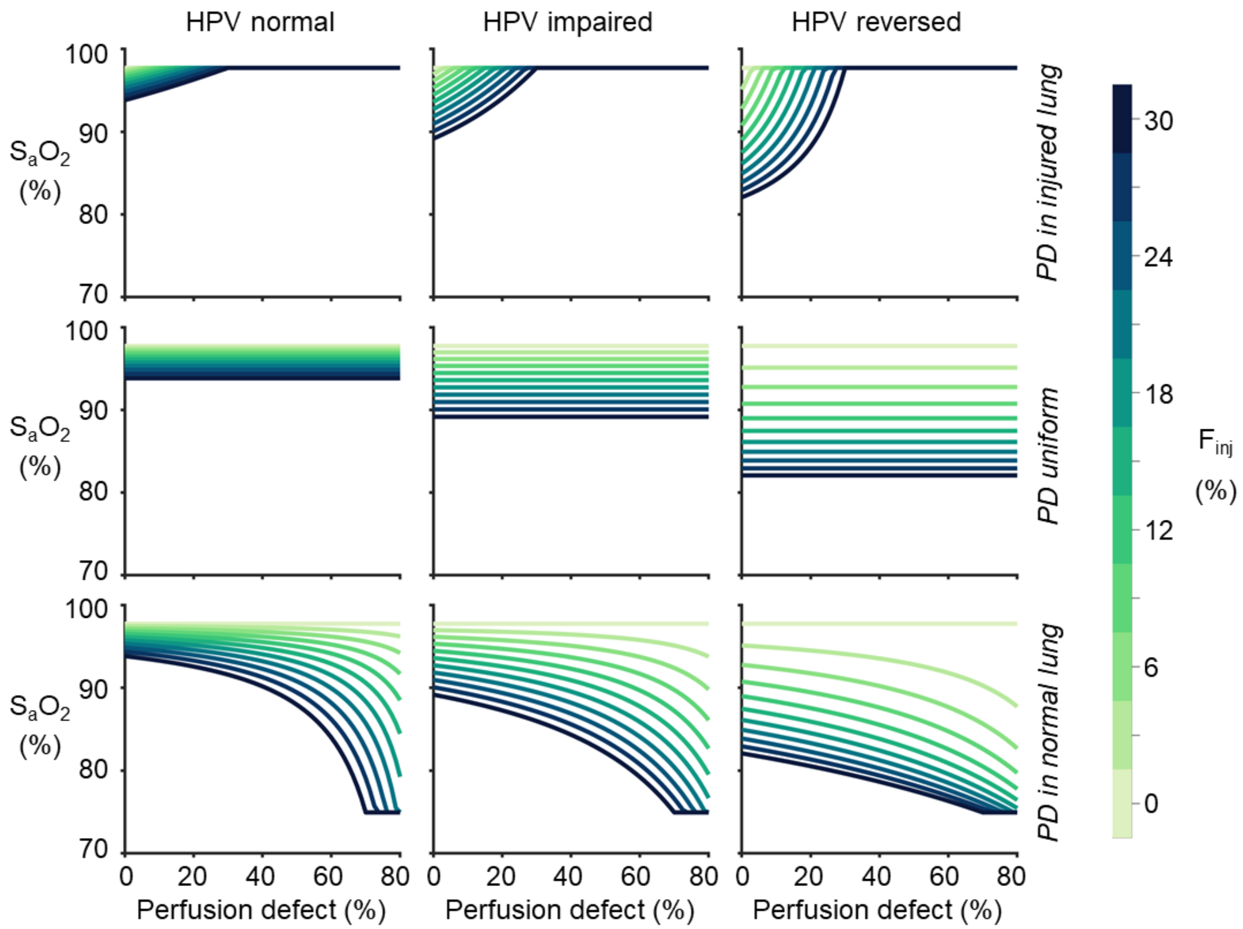

Supplementary Figure 2. Effect of perfusion defect (PD) on arterial oxygen saturation of hemoglobin (S_aO_2) in the early stage model. Columns correspond to alterations in hypoxic pulmonary vasoconstriction (HPV). Rows correspond to the distribution of perfusion defect. Color indicates the fraction of injured lung (F_{inj}). In all cases, F_iO_2 was 21%, mixed venous oxygen tension was 40 mmHg, baseline perfusion gradient was 30%, reversed HPV was modeled with 72% reduction of vascular resistance in injured regions, and there was no assumed ventilation-perfusion mismatching in the noninjured lung.

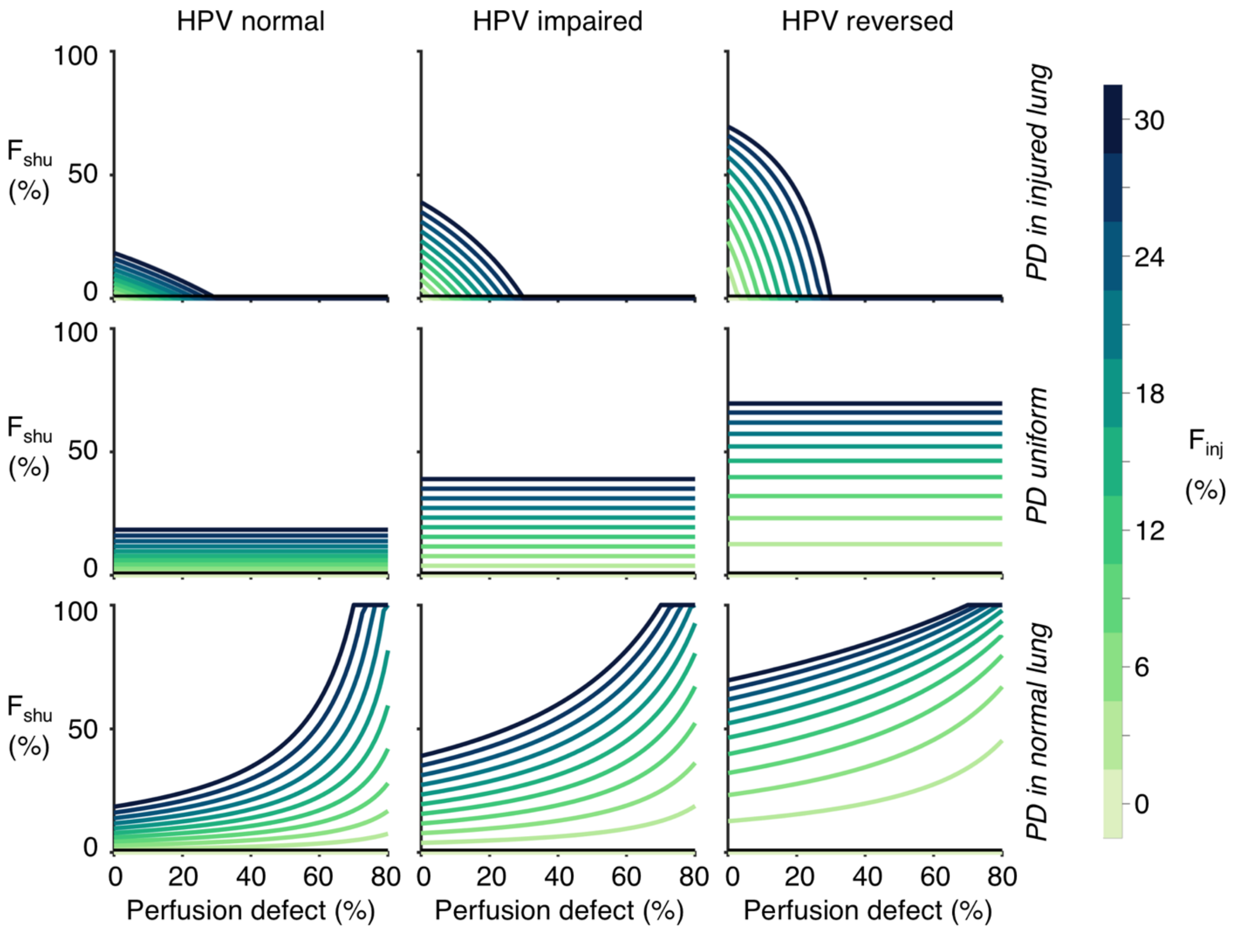

Supplementary Figure 3. Effect of perfusion defect (PD) on the calculated shunt fraction (F_{shu}) in the early stage model. Columns correspond to alterations in hypoxic pulmonary vasoconstriction (HPV). Rows correspond to the distribution of perfusion defect. Color indicates the fraction of injured lung (F_{inj}). In all cases, F_iO_2 was 21%, mixed venous oxygen tension was 40 mmHg, baseline perfusion gradient was 30%, reversed HPV was modeled with 72% reduction of vascular resistance in injured regions, and there was no assumed ventilation-perfusion mismatching in the noninjured lung.

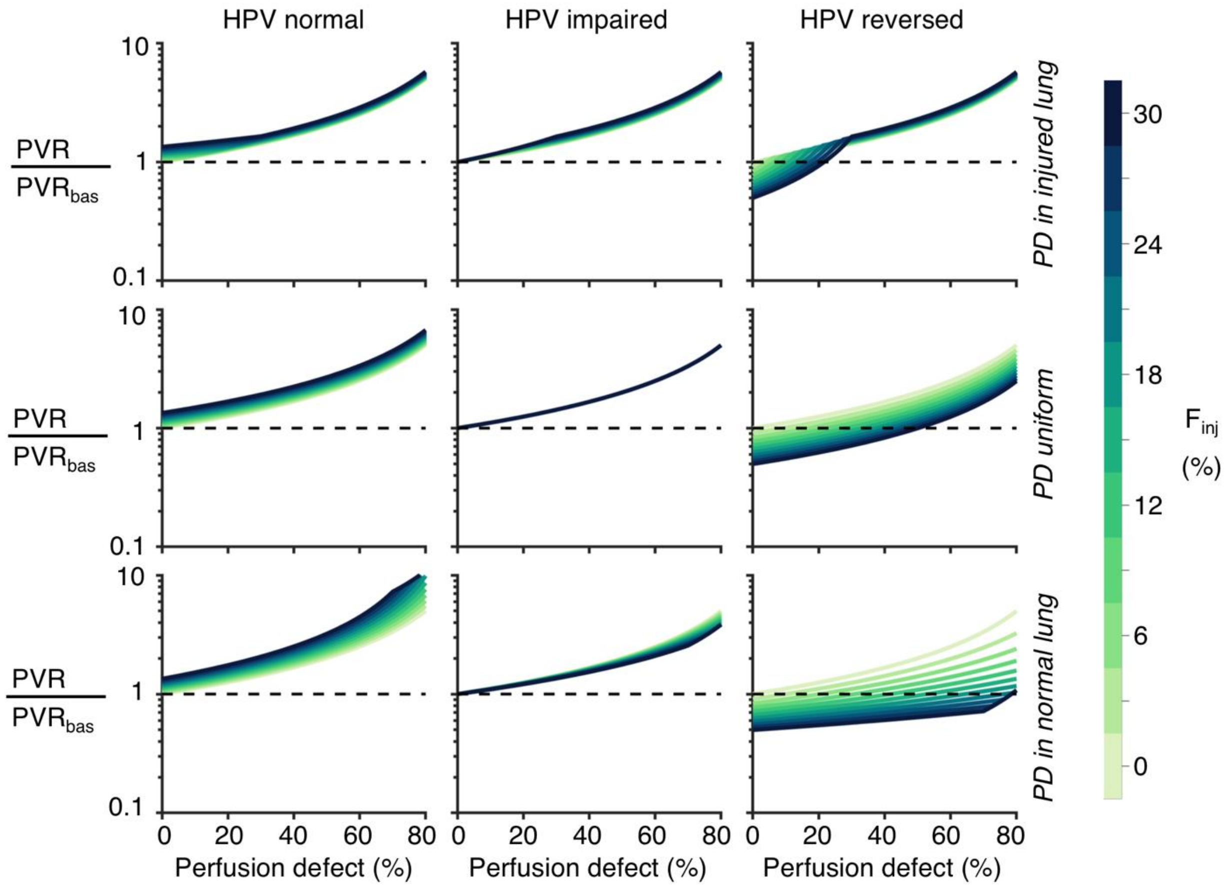

Supplementary Figure 4. Effect of perfusion defect (PD) on the total pulmonary vascular resistance (PVR) relative to the baseline value (PVR_{bas}) in the early stage model. The dashed line indicates no change relative to baseline. Columns correspond to alterations in hypoxic pulmonary vasoconstriction (HPV). Rows correspond to the distribution of perfusion defect. Color indicates the fraction of injured lung (F_{inj}). In all cases, F_iO_2 was 21%, mixed venous oxygen tension was 40 mmHg, baseline perfusion gradient was 30%, reversed HPV was modeled with 72% reduction of vascular resistance in injured regions, and there was no assumed ventilation-perfusion mismatching in the noninjured lung.

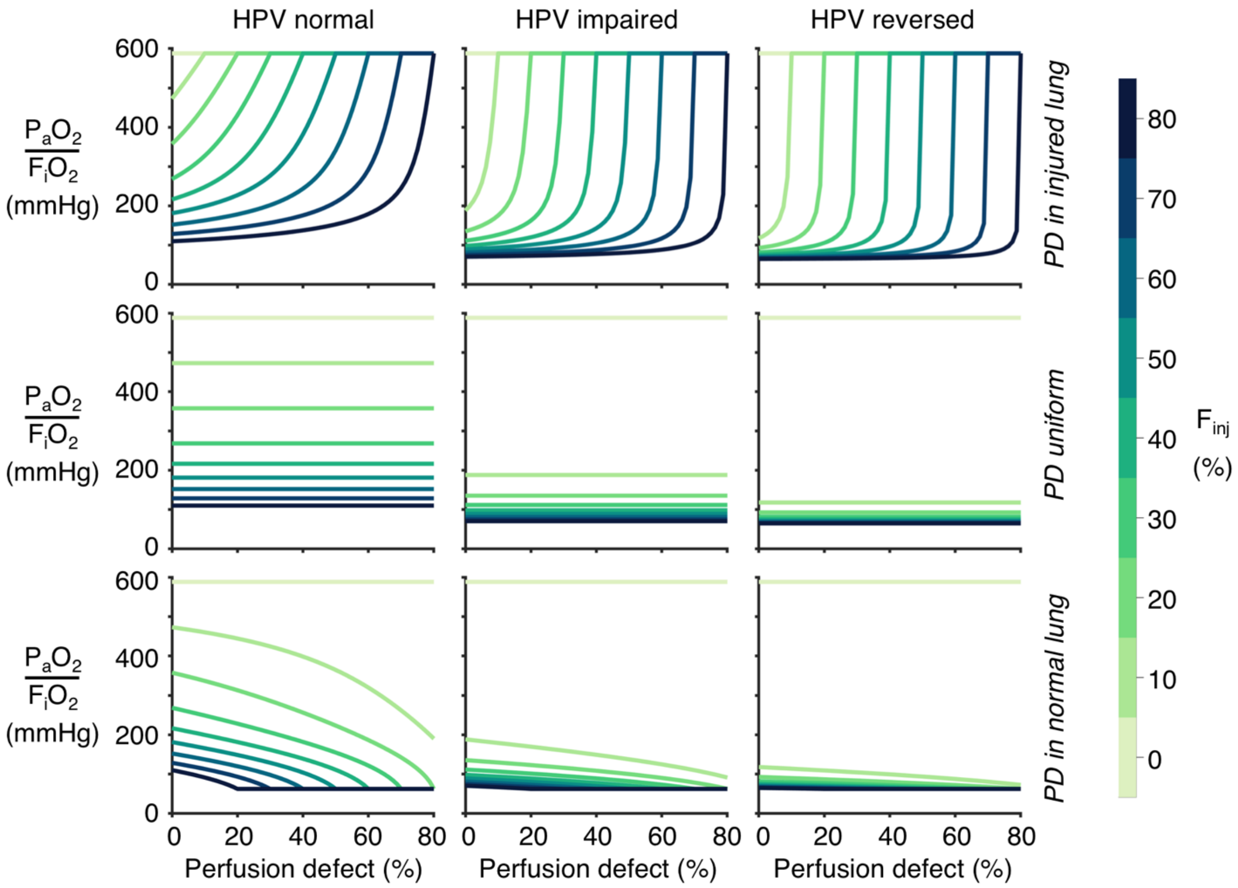

Supplementary Figure 5. Effect of perfusion defect (PD) on the ratio of arterial oxygen tension (P_aO_2) to inspired oxygen fraction (F_iO_2) in the late stage model. Columns correspond to alterations in hypoxic pulmonary vasoconstriction (HPV). Rows correspond to the distribution of perfusion defect. Color indicates the fraction of injured lung (F_{inj}). In all cases, F_iO_2 was 40%, mixed venous oxygen tension was 25 mmHg, baseline perfusion gradient was 30%, reversed HPV was modeled with 72% reduction of vascular resistance in injured regions, and there was no assumed ventilation-perfusion mismatching in the noninjured lung.

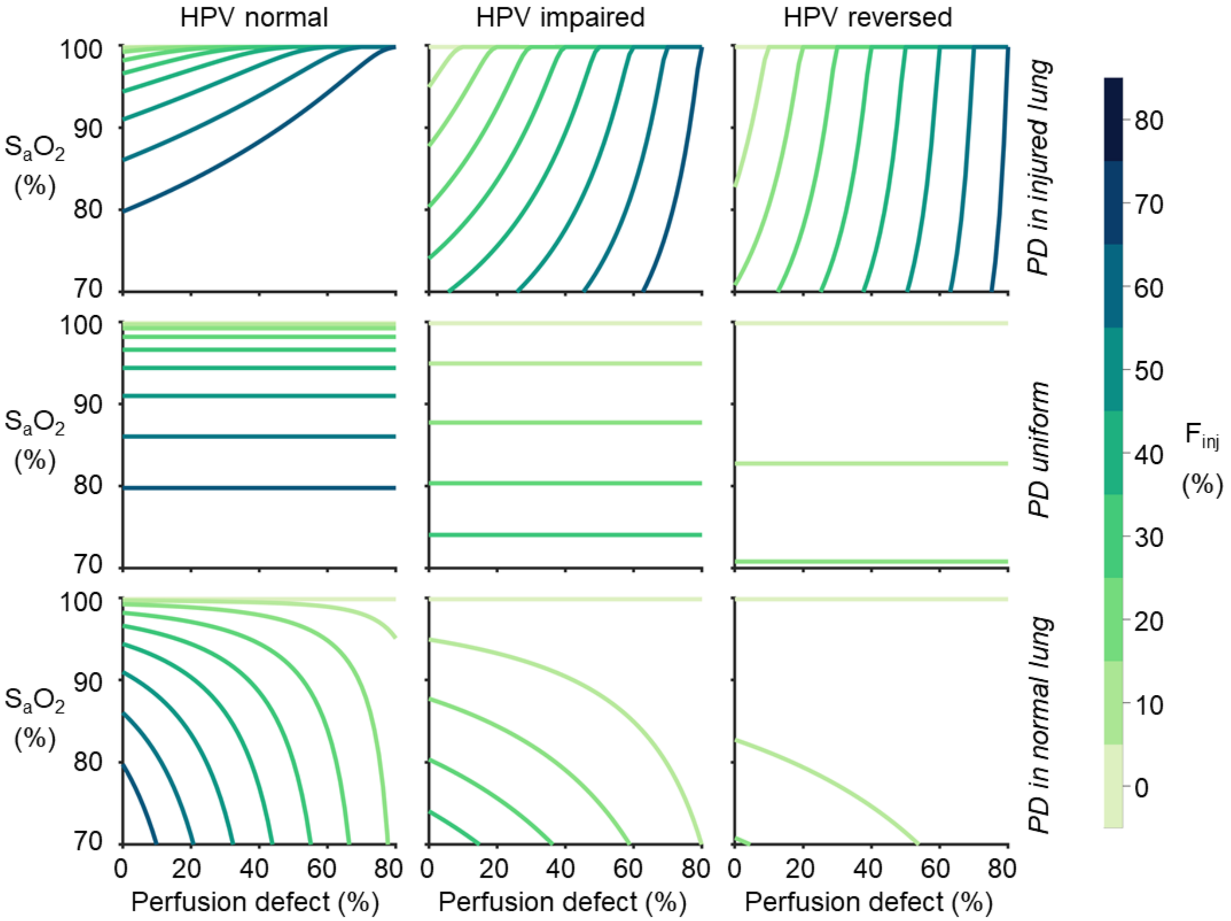

Supplementary Figure 6. Effect of perfusion defect (PD) on arterial oxygen saturation of hemoglobin (S_aO_2) in the late stage model. Columns correspond to alterations in hypoxic pulmonary vasoconstriction (HPV). Rows correspond to the distribution of perfusion defect. Color indicates the fraction of injured lung (F_{inj}). In all cases, F_iO_2 was 40%, mixed venous oxygen tension was 25 mmHg, baseline perfusion gradient was 30%, reversed HPV was modeled with 72% reduction of vascular resistance in injured regions, and there was no assumed ventilation-perfusion mismatching in the noninjured lung. Note that only S_aO_2 above 70% are shown.

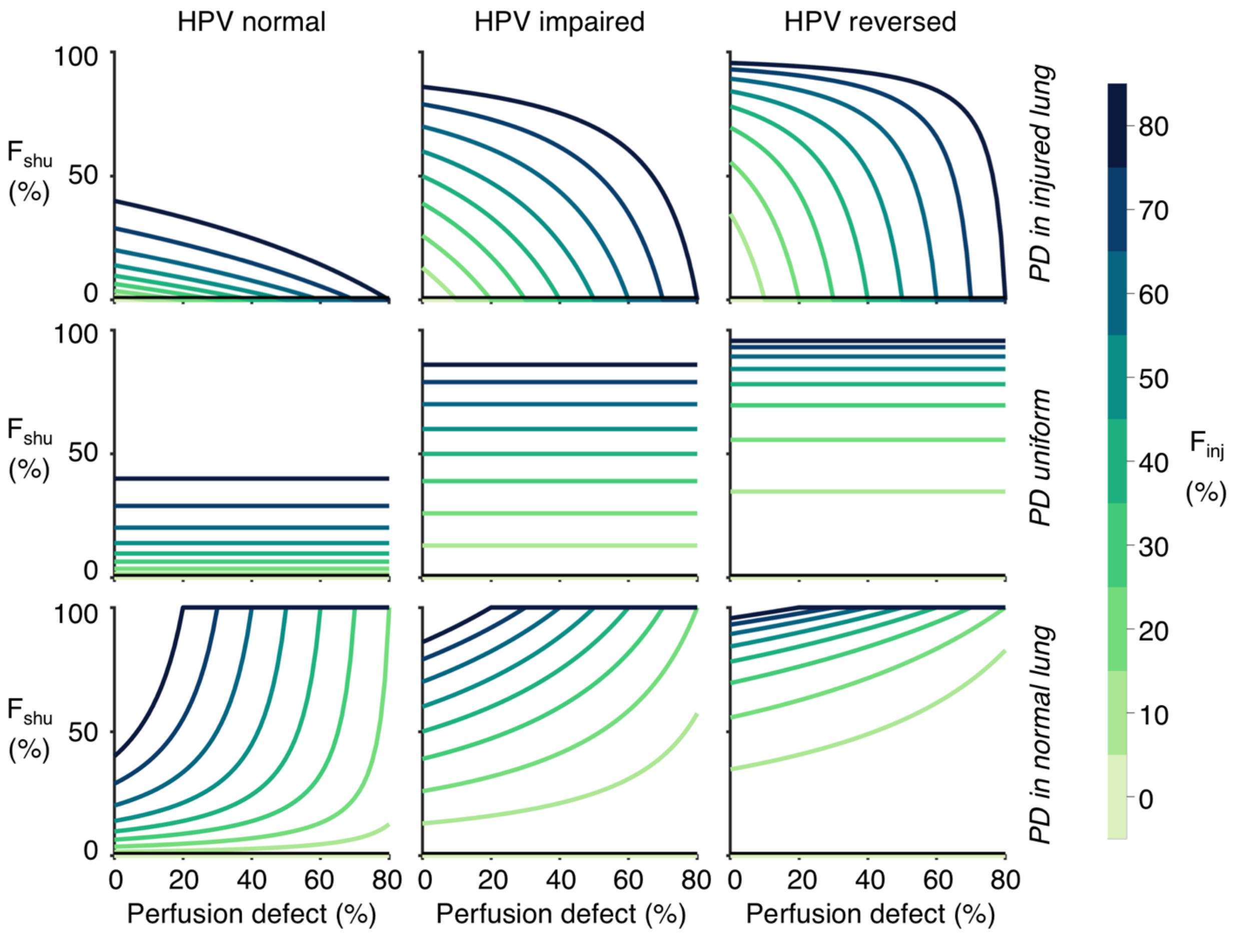

Supplementary Figure 7. Effect of perfusion defect (PD) on calculated shunt fraction (F_{shu}) in the late stage model. Columns correspond to alterations in hypoxic pulmonary vasoconstriction (HPV). Rows correspond to the distribution of perfusion defect. Color indicates the fraction of injured lung (F_{inj}). In all cases, F_iO_2 was 40%, mixed venous oxygen tension was 25 mmHg, baseline perfusion gradient was 30%, reversed HPV was modeled with 72% reduction of vascular resistance in injured regions, and there was no assumed ventilation-perfusion mismatching in the noninjured lung.

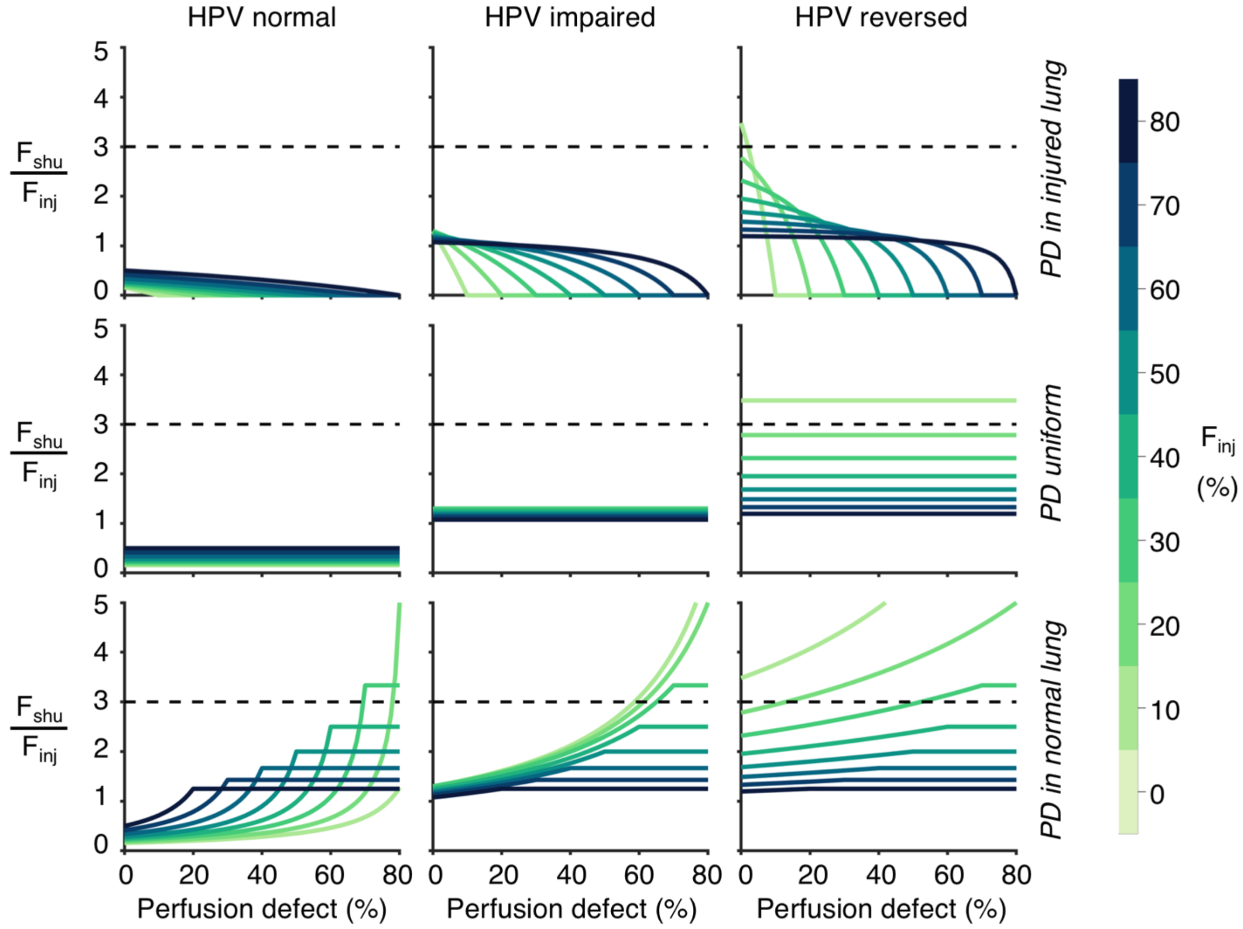

Supplementary Figure 8. Effect of perfusion defect (PD) on the ratio of calculated shunt fraction (F_{shu}) to injured fraction (F_{inj}) in the late stage model. The dashed line indicates a ratio of F_{shu}:F_{inj} = 3, reported in early COVID-19 patients. Columns correspond to alterations in hypoxic pulmonary vasoconstriction (HPV). Rows correspond to the distribution of perfusion defect. Color indicates the fraction of injured lung (F_{inj}). In all cases, F_iO_2 was 40%, mixed venous oxygen tension was 25 mmHg, baseline perfusion gradient was 30%, reversed HPV was modeled with 72% reduction of vascular resistance in injured regions, and there was no assumed ventilation-perfusion mismatching in the noninjured lung.

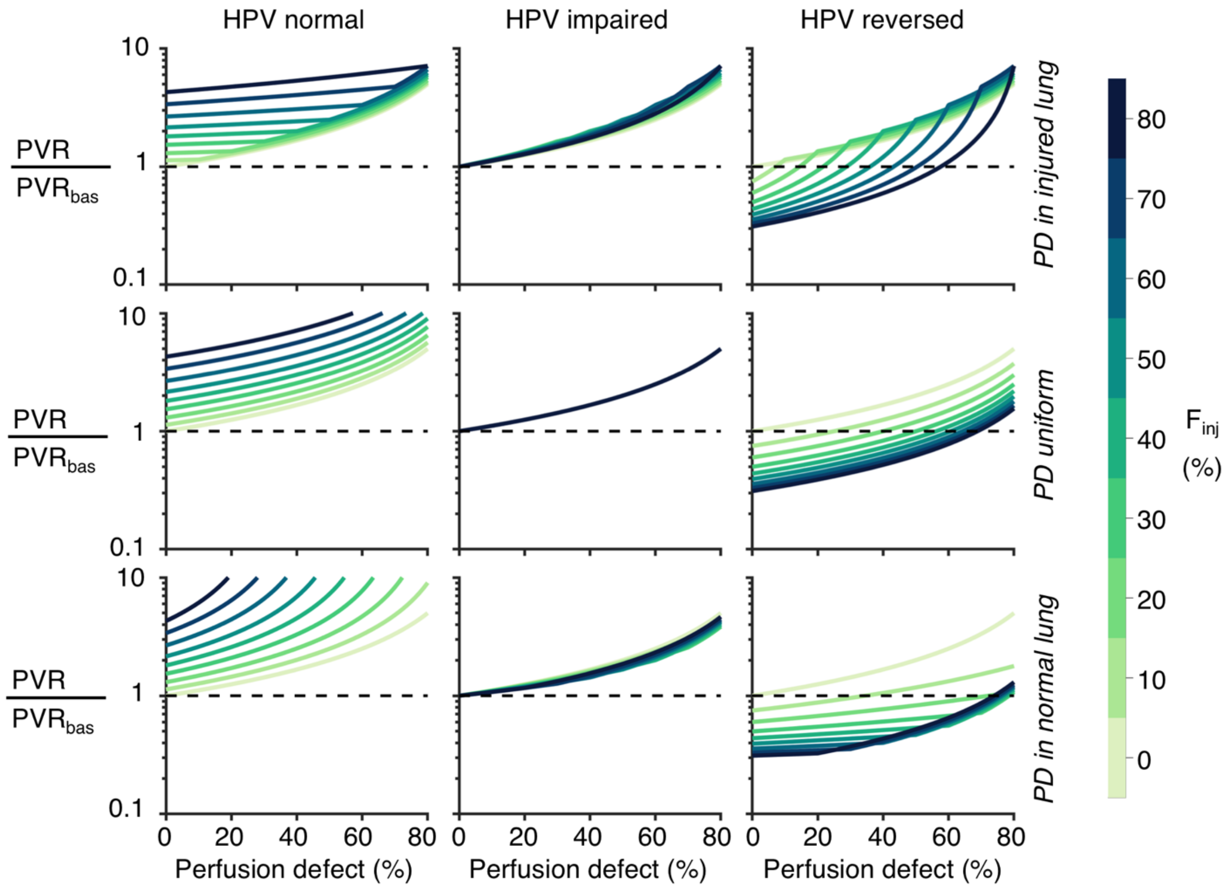

Supplementary Figure 9. Effect of perfusion defect (PD) on the total pulmonary vascular resistance (PVR) relative to the baseline value (PVR_{bas}) in the late stage model. The dashed line indicates no change relative to baseline. Columns correspond to alterations in hypoxic pulmonary vasoconstriction (HPV). Rows correspond to the distribution of perfusion defect. Color indicates the fraction of injured lung (F_{inj}). In all cases, F_iO_2 was 40%, mixed venous oxygen tension was 25 mmHg, baseline perfusion gradient was 30%, reversed HPV was modeled with 72% reduction of vascular resistance in injured regions, and there was no assumed ventilation-perfusion mismatching in the noninjured lung.

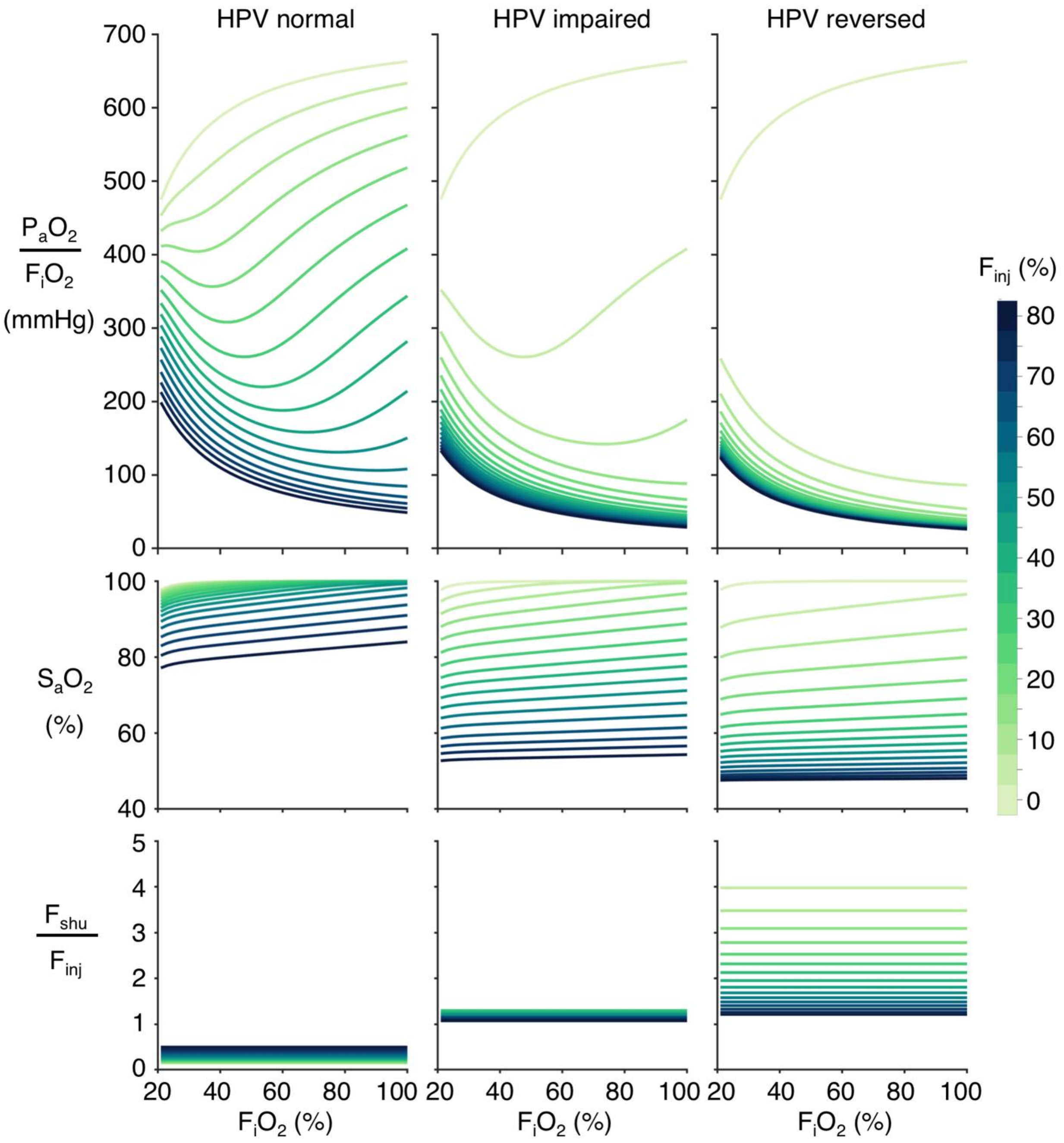

Supplementary Figure 10. Nonlinear dependence of arterial oxygenation on inspired oxygen fraction (F_iO_2) and injured lung fraction (F_{inj}). Columns correspond to type of hypoxic pulmonary vasoconstriction (HPV) modification. Rows correspond to the ratio of arterial oxygen tension to inspired oxygen fraction (P_aO_2:F_iO_2), arterial oxygen saturation of hemoglobin (S_aO_2), and ratio of shunt fraction to injured fraction (F_{shu}:F_{inj}). In all cases, mixed venous oxygen tension (P_vO_2) was 25 mmHg was 17%, F_iO_2 was 60%, baseline perfusion gradient was 30%, and reversed HPV was modeled with 72% reduction of vascular resistance in injured regions.

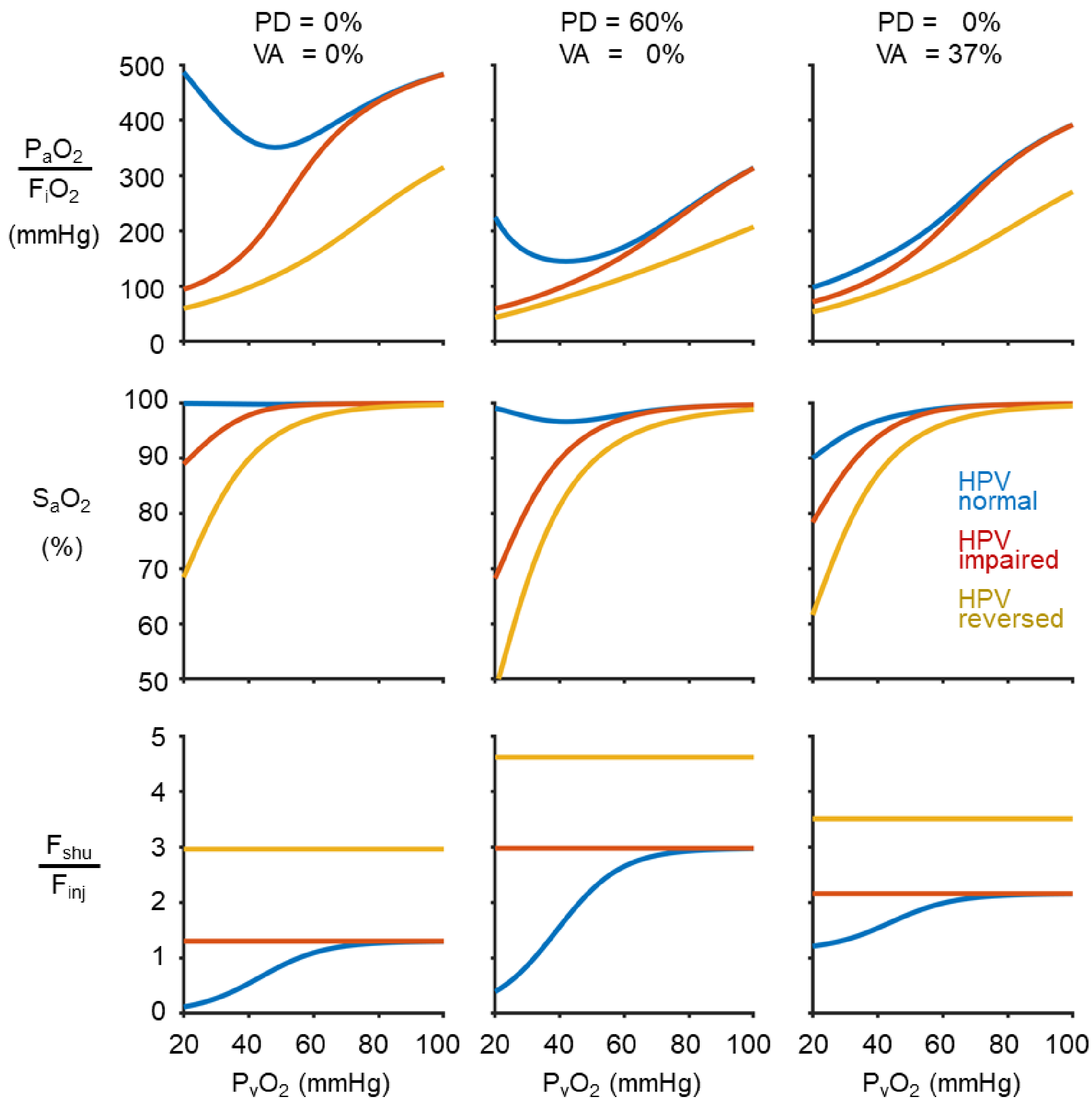

Supplementary Figure 11. Interplay between mixed venous oxygen tension (P_vO_2) and alterations in hypoxic pulmonary vasoconstriction (HPV). Type of HPV modification is indicated by color (blue: normal; red: impaired; yellow: reversed). Rows correspond to the ratio of arterial oxygen tension to inspired oxygen fraction (P_aO_2:F_iO_2), arterial oxygen saturation of hemoglobin (S_aO_2), and ratio of shunt fraction to injured fraction (F_{shu}:F_{inj}). Columns correspond to three different cases of perfusion defect (PD) and venous admixture (VA) affecting the noninjured lung compartments. In all cases, the injured fraction (Finj) was 17%, F_iO_2 was 60%, baseline perfusion gradient was 30%, and reversed HPV was modeled with 72% reduction of vascular resistance in injured regions.

Supplementary Code

<u>*Code Description*</u>

This readme file describes a Matlab script hpv_impairment_model.m written for the purpose of simulating a simple mathematical model of perfusion and gas exchange, with particular emphasis on possible alterations to hypoxic pulmonary vasoconstriction, perfusion defects, and ventilation-perfusion mismatching suspected in patients with the novel coronavirus. The script hpv_impairment_model.m can be executed from the command window. Input parameters at the top of the script file can be modified. Comments next to each input parameter describe its functional meaning in the model. The output is assigned to variables at the bottom of the script file, and can be accessed by name from the command window after executing the script. Comments next to each output variable describe its meaning in the model. The script can be executed dynamically/interactively, or it can be converted into a function for batch evaluation.

Software tested on a computer running a Windows 10 64-bit operating system, with a 3.5 GHz AMD Ryzen 9 3950X CPU and 64 GB RAM. Matlab version R2020a 64-bit was used, including the Optimization Toolbox version 8.5. There is no further installation required. No external data is required. Average execution time for this script was 4 milliseconds on this machine. For the default values that are included with the distributed version of this code, the expected output is given below:

```
F_shunt =
    0.5036

F_shunt_noninjured =
    0.1300

F_shunt_to_F_injured =
    2.9624

PaO2_to_FiO2 =
  248.8098
```

```
PVR =
    1.1826

PaO2 =
   52.2500

SaO2 =
    0.8654
```

Code License

This code is provided under the GNU General Public License version 3 or any later version.

Code

```
% hpv_impairment_model.m
% Model of Gas Exchange with Altered Hypoxic Pulmonary Vasoconstriction,
% Perfusion Defect, and Ventilation-Perfusion Mismatching
% Copyright (C) <2020>  <Jacob Herrmann>
%
% This program is free software: you can redistribute it and/or modify
% it under the terms of the GNU General Public License as published by
% the Free Software Foundation, either version 3 of the License, or
% (at your option) any later version.
%
% This program is distributed in the hope that it will be useful,
% but WITHOUT ANY WARRANTY; without even the implied warranty of
% MERCHANTABILITY or FITNESS FOR A PARTICULAR PURPOSE.  See the
% GNU General Public License for more details.
%
% You should have received a copy of the GNU General Public License
% along with this program.  If not, see <http://www.gnu.org/licenses/>.
%

%______________________________________________________________________%
%____ INPUT PARAMETERS ________________________________________________%

% baseline perfusion gradient (unitless)
% defined as half the range of perfusion divide by the average
Q_baseline_gradient = 0.30 ;

% total fraction of injured lung (unitless)
F_injured = 0.17 ;

% type of injury distribution
% - "lower" focuses injury only in the region with the highest baseline perfusion
% - "middle" focuses injury only in the middle "height" level
% - "upper" focuses injury only in the region with the lowest baseline perfusion
% - "uniform" distribute an equal injured fraction to each "height" level
F_injured_distribution_type = 'lower' ;
```

```
% total fraction of perfusion defect or alveolar deadspace (unitless)
F_deadspace = 0.40 ;

% differential probability of alveolar deadspace occurring in injured
% vs. noninjured comparments (unitless)
F_deadspace_injured = 0.00 ;

% type of hypoxic pulmonary vasoconstriction in the injured
% lung regions.
% - for "normal" function, HPV causes exponentially increasing resistance
%   below a threshold of oxygen tension.
% - for "impaired" function, HPV is absent, there is no response to changes
%   in oxygen tension.
% - for "reversed" function, HPV is not only absent, but instead there is a
%   reduction in resistance in injured regions, regardless of oxygenation
hpv_type = 'reversed' ;

% fractional reduction of vascular resistance in the injured
% lung regions (unitless). note that this parameter only applies
% to the HPV reversed model.
injured_R_reduction = 0.50 ;

% fractional equilibration between alveolar and end-capillary
% gas tensions in the injured and noninjured lung regions (unitless).
O2_equilibration_injured = 0.05 ;

% venous admixture (shunt fraction) in blood leaving the noninjured lung
% compartments, resulting from ventilation-perfusion mismatching. note that
% this specified value is only effective at 21% inspired oxygen.  the effective
% noninjured venous admixture will be linearly reduced to zero as inspired
% oxygen fraction increases from 21% to 100%.
F_shunt_noninjured_21 = 0.13 ;

%______________________________________________________________________%
%____ OTHER PARAMETERS ________________________________________________%

% fraction of inspired oxygen (unitless)
FiO2 = 0.21 ;

% partial pressure of oxygen in mixed venous blood (mmHg)
PvO2 = 40 ;

% water vapor partial pressure (mmHg)
PH2O = 47 ;

% partial pressure of carbon dioxide in arterial blood (mmHg)
PaCO2 = 40 ;

% respiratory quotient (unitless)
respiratory_quotient = 0.8 ;

% hemoglobin concentration in blood (g /dL)
hemoglobin = 14 ;
```

```
%_____________________________________________________________________%
%____ MATHEMATICAL MODEL ______________________________________________%

% solve for the average steady state partial pressure of oxygen
% in normal alveolar gas using the alveolar gas equation.
PAO2 = FiO2*(760-PH2O) - (PaCO2/respiratory_quotient) ;

% function for oxygen saturation of hemoglobin (unitless),
% given input parameter partial pressure of oxygen
% using a regression provided by Severinghaus 1979.
% https://www.ncbi.nlm.nih.gov/pubmed/35496
S_O2 = @(P_O2) 1./( 1 + 23400./(power(P_O2,3)+150*P_O2) ) ;

% oxygen content equation (dL O2 /dL blood), also
% provided by Severinghaus 1979.
% https://www.ncbi.nlm.nih.gov/pubmed/35496
C_O2 = @(P_O2,S_O2) bsxfun(@plus, 1.34*hemoglobin*S_O2 , 0.0031*P_O2 ) ;

% oxygen saturation and blood content in mixed-venous blood and
% in end-capillary blood blood from an ideal well-aerated
% compartment of the lung
SvO2 = S_O2( PvO2 ) ;
CvO2 = C_O2( PvO2 , SvO2 ) ;
ScO2_aerated = S_O2( PAO2 ) ;
CcO2_aerated = C_O2( PAO2 , ScO2_aerated ) ;

% linearly reduce venous admixture caused by V/Q mismatching as
% inspired oxygen fraction increases from 21% to 100%
F_shunt_noninjured = F_shunt_noninjured_21 * (1.00-FiO2)/(1.00-0.21) ;

% find the fractional equilibration of oxygen required to
% produce a specified amount of venous admixture specifically from
% the noninjured lung compartment
PcO2_noninjured = @(B) PvO2 + B*(PAO2-PvO2) ;
CcO2_noninjured = @(B) C_O2( PcO2_noninjured(B) , S_O2(PcO2_noninjured(B)) ) ;
Fshu_noninjured_estimate = @(B) (CcO2_aerated-CcO2_noninjured(B)) / ...
      (CcO2_aerated-CvO2) ;
error_Fshu_noninjured = @(B) power( Fshu_noninjured_estimate(B) - ...
      F_shunt_noninjured ,2) ;
options = optimoptions('fmincon','Display','none') ;
O2_equilibration_noninjured = fmincon( ...
      error_Fshu_noninjured , ...
      1.0-F_shunt_noninjured , ...
      [],[],[],[],0.0,1.0,[],options) ;

% determine the injured fraction of each "height" level, based on
% the total fractional lung injury and the type of injury distribution.
switch F_injured_distribution_type
      case 'lower'   ; F_injured_height = [0,0,1]' ;
      case 'middle'  ; F_injured_height = [0,1,0]' ;
      case 'upper'   ; F_injured_height = [1,0,0]' ;
      case 'uniform' ; F_injured_height = [1,1,1]'/3 ;
end
F_injured_height = 3 * F_injured_height * F_injured ;
F_injured_height = max(0,min(1, F_injured_height )) ;
```

```
if abs( F_injured - sum(F_injured_height)/3 ) > 0.01
      disp(['Warning: More than 1% deviation between specified injured fraction' ...
            ' and sum of regional injured fractions.'])
end

% define the regional response of hypoxic pulmonary vasoconstriction
% within the injured compartments of the model.
% - for "normal" function, HPV causes exponentially increasing resistance
%   below a threshold of oxygen tension.
% - for "impaired" function, HPV is absent, there is no response to changes
%   in oxygen tension.
% - for "reversed" function, HPV is not only absent, but instead there is a
%   reduction in resistance in injured regions, regardless of oxygenation
hpv_threshold = 50 ;
switch hpv_type
      case 'normal'   ; hpv = @(x,injured) 1 + 100.*exp(-x/(0.2*hpv_threshold)) ;
      case 'impaired' ; hpv = @(x,injured) ones(size(x)) ;
      case 'reversed' ; hpv = @(x,injured) ones(size(x)) - (1-
injured_R_reduction)*injured ;
end

% determine how to partition the alveoalr deadspace, with a differential
% probability of occuring in injured vs. noninjured comparments.  Note that
% the total deadspace fraction will be ensured, but these probabilities may
% not be preserved.  A warning will be displayed.
if (F_injured<eps()) || (F_deadspace_injured<eps())
      tmp2 = 0.0 ;
      tmp1 = 1.0 ;
else
      ratio = ((1-F_deadspace_injured)/F_deadspace_injured) * ((1-
F_injured)/F_injured) ;
      tmp2 = 1/(1+ratio) ;
      tmp1 = 1 - tmp2 ;
end
if (tmp1*F_deadspace) > (1-F_injured)
      disp('Warning: Deadspace could not be split as desired.')
      tmp2 = tmp2 + (tmp1-(1-F_injured)/F_deadspace) ;
      tmp1 = (1-F_injured)/F_deadspace ;
elseif (tmp2*F_deadspace) > F_injured
      disp('Warning: Deadspace could not be split as desired.')
      tmp1 = tmp1 + (tmp2-F_injured/F_deadspace) ;
      tmp2 = F_injured/F_deadspace ;
end
F_deadspace_compartment = F_deadspace * [ tmp1 , tmp2 ] ;
clear tmp1 tmp2

% fraction of total lung represented by each of the 12 compartments (unitless).
% - rows (1st index) correspond to "height" levels
% - columns (2nd index) correspond to (noninjured, injured) compartments
% - layers (3rd index) correspond to (perfused, deadspace) compartments
tmp3 = (1/3) * cat(2, 1-F_injured_height , F_injured_height ) ;
if F_injured < eps()
      tmp4 = tmp3 ;
elseif (1-F_injured) < eps()
      tmp4 = tmp3 ;
```

```
else
      tmp4 = bsxfun(@rdivide, tmp3 , [1-F_injured,F_injured] ) ;
end
tmp5 = bsxfun(@times, tmp4 , F_deadspace_compartment ) ;
F_compartment = cat(3, tmp3-tmp5 , tmp5 ) ;
clear tmp3 tmp4 tmp5

% boolean value representing whether a compartment is injured (true)
% or normal (false).  rows, columns, and layers have the same meaning as F.
injured = repmat( [false,true] ,[3,1,2]) ;

% equilibration between alveolar and end-capillary gas tension (unitless).
% - normal compartment may have some venous admixture due to V/Q mismatching.
% - injured compartment has little or no equilibration.
O2_equilibration = (~injured*O2_equilibration_noninjured) +
(injured*O2_equilibration_injured) ;

% end-capillary partial pressure of oxygen in each compartment (mmHg)
% is assumed to be a weighted average of mixed venous oxygen tension
% and alveolar oxygen tension.
PcO2  = PvO2 + O2_equilibration*( PAO2 - PvO2 ) ;

% baseline perfusion as a fraction of cardiac output (unitless).
% baseline perfusion is computed to ensure a specified baseline
% perfusion gradient, BEFORE effects of HPV are considered.
% there is only one column, corresponding to each "height" level
% before injury.
Q_baseline = 1/3 ...
      + linspace(0,Q_baseline_gradient*2/3,3)' ...
      - (0.5*Q_baseline_gradient*2/3) ;

% normalize pulmonary vascular resistance to baseline value (unitless)
PVR_baseline = 1 ;

% baseline resistance at each height level.
% these values are determined by the baseline perfusion gradient.
% note that this quantity represents a normalized resistance.
R_baseline = (PVR_baseline./Q_baseline) ;

% resistance in each compartment after modifications (unitless),
% accounting for effects of altered or normal HPV in injured compartments.
R_modified = bsxfun(@times, R_baseline , hpv(PcO2,injured) ) ;

% set vascular resistance of deadspace compartments to infinite
R_modified(:,:,2) = inf ;

% resistance in each compartment (unitless), accounting for fraction
% of lung represented by each compartment.  note that a compartment
% representing 0% of the lung will exhibit infinite resistance, ensuring
% that no perfusion is received.
R = R_modified .* ((1/3)/F_compartment) ;

% total pulmonary vascular resistance (unitless), normalized by baseline PVR
PVR = 1/sum(1./R(:)) ;
```

```matlab
% perfusion to each compartment (unitless), as a fraction of cardiac output
Q = (PVR./R) ;

% evaluate oxygen saturation and content equations for end-capillary blood
% in each compartment.
ScO2 = S_O2( PcO2 ) ;
CcO2 = C_O2( PcO2 , ScO2 ) ;

% compute oxygen content in mixed arterial blood by a perfusion-weighted
% average of end-capillary oxygen contents across all compartments.
CaO2 = sum(reshape( Q.*CcO2 ,[],1)) ;

% numerically approximate oxygen tension in mixed arterial blood, by ensuring
% that the result is consistent with the computed arterial oxygen content.
error_CaO2 = @(P) power( CaO2 - (C_O2(P,S_O2(P))) ,2) ;
guess_PaO2 = PvO2 ;
PaO2 = fminsearch( error_CaO2 , guess_PaO2 ) ;
SaO2 = S_O2( PaO2 ) ;

%______________________________________________________________________%
%____ OUTPUT VARIABLES ________________________________________________%

% return the pulmonary shunt fraction (unitless).
% this represents the fraction of cardiac output which is nonoxygenated.
% note that this calculation compares mixed arterial oxygen context to
% the oxygen content in an ideal well-aerated normal compartment.
F_shunt = (CcO2_aerated-CaO2)/(CcO2_aerated-CvO2) ;

% return the PAO2:FiO2 ratio (mmHg).
% this represents the severity of hypoxemia.
PaO2_to_FiO2 = PaO2 / FiO2 ;

% return the Fshu:Finj ratio (unitless)
F_shunt_to_F_injured = F_shunt / F_injured ;

% display to command window
F_shunt
F_shunt_noninjured
F_shunt_to_F_injured
PaO2_to_FiO2
PVR
PaO2
SaO2
```
